# Supplementary figures and images for: Executive Function Response to Moderate-to-High-Intensity Rope Skipping in Overweight Adolescents Aged 12–14: A Preliminary Study
Source: J Funct Morphol Kinesiol. 2025 Apr 29;10(2):152. doi: 10.3390/jfmk10020152 (PMC12101173; doi:10.3390/jfmk10020152)

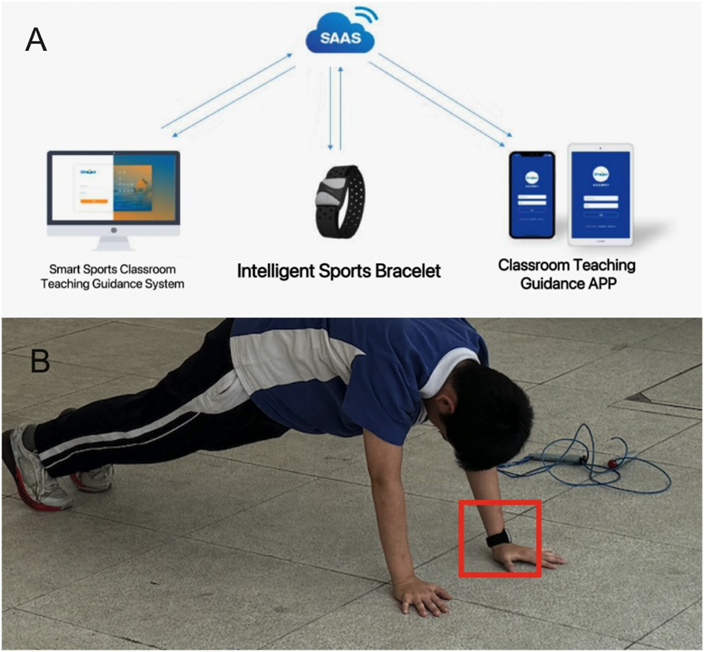

Supplement: Supplementary file 1 [file jfmk-10-00152-s001.zip › Figure S1-Appendix A. The intelligent sports classroom exercise load guidance system fitness trackers.tif]
